# ERAP2 Is Associated With Immune Infiltration and Predicts Favorable Prognosis in SqCLC

**DOI:** 10.3389/fimmu.2021.788985

**Published:** 2021-12-21

**Authors:** Zhenlin Yang, He Tian, Fenglong Bie, Jiachen Xu, Zheng Zhou, Junhui Yang, Renda Li, Yue Peng, Guangyu Bai, Yanhua Tian, Ying Chen, Lei Liu, Tao Fan, Chu Xiao, Yujia Zheng, Bo Zheng, Jie Wang, Chunxiang Li, Shugeng Gao, Jie He

**Affiliations:** ^1^ Department of Thoracic Surgery, National Cancer Center/National Clinical Research Center for Cancer/Cancer Hospital, Chinese Academy of Medical Sciences, Peking Union Medical College, Beijing, China; ^2^ Department of Medical Oncology, National Cancer Center/National Clinical Research Center for Cancer/Cancer Hospital, Chinese Academy of Medical Sciences, Peking Union Medical College, Beijing, China; ^3^ Genetron Health (Beijing) Co. Ltd., Beijing, China; ^4^ Department of Thoracic Surgery/Head & Neck Medical Oncology, The University of Texas (UT) MD Anderson Cancer Center, Houston, TX, United States; ^5^ Department of Thoracic Surgery I, The Third Affiliated Hospital of Kunming Medical University (Yunnan Cancer Hospital, Yunnan Cancer Center), Kunming, China; ^6^ Department of Pathology, National Cancer Center/National Clinical Research Center for Cancer/Cancer Hospital, Chinese Academy of Medical Sciences, Peking Union Medical College, Beijing, China

**Keywords:** ERAP2, squamous cell lung cancer (SqCLC), prognosis, immune microenvironment, biomarker

## Abstract

**Background:**

Immunotherapy has been proven effective among several human cancer types, including Squamous cell lung carcinoma (SqCLC). ERAP2 plays a pivotal role in peptide trimming of many immunological processes. However, the prognostic role of ERAP2 and its relationship with immune cell infiltration in SqCLC remains unclear.

**Methods:**

The differential expression of ERAP2 was identified *via* GEO and TCGA databases. We calculated the impact of ERAP2 on clinical prognosis using the Kaplan-Meier plotter. TIMER was applied to evaluate the abundance of immune cells infiltration and immune markers. SqCLC tissue microarrays containing 190 patients were constructed, and we performed immunohistochemical staining for ERAP2, CD8, CD47, CD68, and PD-L1 to validate our findings in public data.

**Results:**

In the GEO SqCLC database, ERAP2 was upregulated in patients with better survival (p=0.001). ERAP2 expression in SqCLC was significantly lower than that of matched normal samples (p<0.05) based on TCGA SqCLC data. Higher expression of ERAP2 was significantly associated with better survival in SqCLC patients from TCGA (p=0.007), KM-plotter (p=0.017), and our tissue microarrays (TMAs) (p=0.026). In univariate and multivariate Cox analysis of SqCLC TMAs, high ERAP2 expression was identified as an independent protective factor for SqCLC patients (Univariate Cox, HR=0.659, range 0.454-0.956, p<0.05. Multivariate Cox, HR=0.578, range 0.385-0.866, p<0.05). In TIMER, ERAP2 was positively correlated with several immune markers (CD274, p=1.27E-04; CD68, p=5.88E-08) and immune infiltrating cells (CD8^+^ T cell, p=4.09E-03; NK cell, p=1.00E-04). In our cohort, ERAP2 was significantly correlated with CD8^+^ tumor-infiltrating lymphocytes (TILs) (p=0.0029), and patients with higher ERAP2 expression had a higher percentage of PD-L1 positive patients (p=0.049) and a higher CD8^+^ TILs level (p=0.036).

**Conclusions:**

For the first time, our study demonstrates that higher expression of ERAP2 is tightly associated with the immuno-supportive microenvironment and can predict a favorable prognosis in SqCLC. Meanwhile, ERAP2 may be a promising immunotherapeutic target for patients with SqCLC.

## Introduction

According to the newest data published in 2021, lung cancer has the highest mortality rate among all cancer types and is a significant health care concern throughout the world (1). Approximately 85% of lung cancer can be classified into a histological subtype generally known as non-small cell lung cancer (NSCLC), 25% to 30% of which is squamous cell lung cancer (SqCLC) (2). SqCLC had specific clinicopathologic characteristics, including male gender, older age, smoker preference, comorbidities, and centrally located tumors (3). Patients with SqCLC are usually diagnosed at an advanced stage, and the 5-year overall survival rate of advanced SqCLC was less than 20%.

Even though standard platinum-based chemotherapy is the mainstay of first-line treatment for most SqCLC patients (4), there are still many patients who cannot receive satisfying outcomes (5). Additionally, different from lung adenocarcinoma (LUAD), which has greatly benefited from targeted therapies against driver mutations such as epidermal growth factor receptor (EGFR) mutations, etc., inroads in targeted therapy are rare in SqCLC (6, 7). Over the past few decades, the limited therapeutic options rendered SqCLC a challenging-to-treat disease. The introduction of immunotherapy, particularly blockers of the PD-1 axis, into the treatment of NSCLC has revolutionized the therapeutic stand-care of this recalcitrant disease, yielding significant survival benefits (8). However, only a minority of SqCLC patients have achieved sustained benefits. Primary and acquired resistances are common phenomena in SqCLC immunotherapy (9). The immunotherapy of SqCLC is still in its infancy, and more immune-related treatments are warranted.

ERAP2 (Endoplasmic Reticulum Aminopeptidase 2), located on chromosome 5q15, belongs to the oxytocinase subfamily of M1 aminopeptidases and is closely related to ERAP1(endoplasmic Reticulum Aminopeptidase 1), a homologous enzyme of it (10). Both ERAP2 and ERAP1 mainly participate in the final trimming of peptides that will be loaded on MHC-I (Major Histocompatibility Complex class I) molecules at the cell surface for CD8^+^ T cells/Natural Killer (NK) cells recognition (11, 12). The length of some peptides entering ER is not suitable for being loaded onto MHC I molecule and thus will be trimmed by ERAP1/2 (13). In the immune-evading tumor, malfunction ERAP2 can undermine tumor-associated antigenic episodes while the immune checkpoints are over-expressed, thus anti-tumor T cell response is suspended (14). Previous studies have highlighted that ERAPs are potential targets for enhancing T/NK cell-mediated immunogenicity of malignant cells for developing anti-tumor immunotherapy (15, 16). In addition, Lim et al. has proved that in bladder cancer patients receiving anti-PD-L1 therapy, ERAP2 expression can stratify overall survival (17). The deficiencies in the expression and function of ERAPs have been demonstrated in multiple tumor types, including lung cancer (18–20). So far, the biological roles of ERAP2 in SqCLC have remained unclear.

This study explored the predictive value and immune-related roles of ERAP2 in SqCLC using public databases. We validated our conclusions in a large-scale SqCLC tissue-microarray with long-term follow-up data. We aimed to illustrate the biological functions of ERAP2 in SqCLC and provide novel clues for administering immunotherapy in SqCLC.

## Materials and Methods

### Bioinformatic Mining of ERAP2

**Figure 1:**
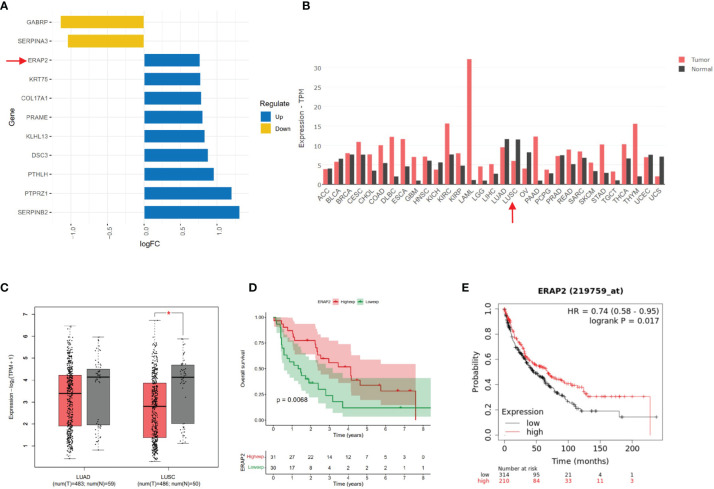
Bioinformatic mining of ERAP2. **(A)** High and low expression genes of SqCLC patients with better survival based on GEO dataset GSE30219. The length of the bar represents the log (fold change) of gene expression between patients with different survival. Orange, low expression genes in patients with better survival. Blue, high expression genes in patients with longer survival. **(B)** ERAP2 expression profiles across all 31 tumor types and paired normal tissues based on TCGA data. The height of the bar represents the median expression of certain tumor types or normal samples. Red, tumor; Black, normal tissue. Arrow, ERAP2 expression in SqCLC. ACC, Adrenocortical carcinoma; BLCA, Bladder Urothelial Carcinoma; BRCA, Breast invasive carcinoma; CESC, Cervical squamous cell carcinoma and endocervical adenocarcinoma; CHOL, Cholangiocarcinoma; COAD, Colon adenocarcinoma; DLBC, Lymphoid Neoplasm Diffuse Large B-cell Lymphoma; ESCA, Esophageal carcinoma; GBM, Glioblastoma multiforme; HNSC, Head and Neck squamous cell carcinoma; KICH, Kidney Chromophobe; KIRC, Kidney renal clear cell carcinoma; KIRP, Kidney renal papillary cell carcinoma; LAML, Acute Myeloid Leukemia; LGG, Brain Lower Grade Glioma; LIHC, Liver hepatocellular carcinoma; LUAD, Lung adenocarcinoma; LUSC, Lung squamous cell carcinoma; OV, Ovarian serous cystadenocarcinoma; PAAD, Pancreatic adenocarcinoma; PCPG, Pheochromocytoma and Paraganglioma; PRAD, Prostate adenocarcinoma; READ, Rectum adenocarcinoma; SARC, Sarcoma; SKCM, Skin Cutaneous Melanoma; STAD, Stomach adenocarcinoma; TGCT, Testicular Germ Cell Tumors; THCA, Thyroid carcinoma; THYM, Thymoma; UCEC, Uterine Corpus Endometrial Carcinoma; USC, Uterine Carcinosarcoma. **(C)** ERAP2 expression in LUAD and LUSC based on data in GEPIA. Left, LUAD. Right, LUSC. Red, tumor sample. Grey, normal sample. Asterisk, p value<0.05; Centerline, median; box limits, upper and lower quartiles; points, outliers. **(D)** K-M curve showing the overall survival of SqCLC patients stratified by ERAP2 expression in the GEO dataset. Red, ERAP2 high expression group. Green, ERAP2 low expression group. **(E)** K-M curve showing the overall survival of SqCLC patients stratified by ERAP2 expression in the KM-Plotter dataset. Red, ERAP2 high expression group. Black, ERAP2 low expression group.

Using R software, we obtained differentially expressed genes (DEGs) from patients with distinct survival in the GEO dataset (GSE30219). GSE30219 dataset contained 61 SqCLC patients, whose median survival time (59 months) was used to stratify groups with better and worse survival. Totally 11 DEGs (p<0.05) of the two groups were extracted using the R package limma. The list of DEGs and corresponding logFC values were shown in **Figure 1A**. Using GEPIA (http://gepia2.cancer-pku.cn/#index), an online analysis website containing pan-cancer transcriptome data from TCGA (https://portal.gdc.cancer.gov/), we explored the ERAP2 expression level among multiple cancer types and paired normal samples (**Figure 1B**), in which the SqCLC dataset included 486 tumor samples and 338 normal samples (Red Arrow in **Figure 1B**).Using patients with follow-up data from the GEO dataset (GSE30219), we performed survival analysis in SqCLC stratified by ERAP2 expression. KM-plotter (http://www.kmplot.com/lung/) was used to validate the prognostic effect of ERAP2 in SqCLC that we found in the GEO dataset.

### Tissue Microarray Construction and IHC Staining

Following the IRB (Institutional Review Board) approval, formalin-fixed, paraffin-embedded tissue microarrays (TMAs) were created using SqCLC samples collected from patients who underwent surgery from April 2010 to August 2011 in the Department of Thoracic Surgery, Cancer Hospital, Chinese Academy of Medical Sciences and Peking Union Medical College. The TMAs contained 190 SqCLC tumor samples. The tumor tissues were fixed by formalin and embedded in paraffin. We took two 2-mm cores from each sample to constitute the TMAs, and then 4-μm thick TMA sections were manufactured. All manual process was conducted by professional pathological technicians from the Department of Pathology of our hospital. All specimens in the 3 TMAS were diagnosed, selected, and confirmed by two certified pathological clinicians.

We performed immunohistochemistry (IHC) of several markers on the TMA, including ERAP2, PD-L1, CD47, CD8, and CD68. We incubated the TMAs with the primary antibodies against ERAP2 (Sigma, HPA034498), PD-L1 (Abcam, 28-8), CD47 (Abcam, EPR21794), CD8 (CST, D8A8Y), and CD68 (Abcam, KP1), and then with the secondary antibodies and 3, 3’-diaminobenzidine (DAB).

Two independent pathologists without prior knowledge of our research evaluated the IHC staining results. ERAP2 expression was scored using a combined method (21). Negative, weak, moderate, and strong intensities were scored as 0, 1, 2, and 3, respectively. The percentage of cells that stained at each intensity score was estimated visually. The ultimate score for each specimen was calculated as the sum of the percentage of stained cells multiplied by the intensity scores. For instance, a sample with 10% negative staining, 40% moderate staining, and 50% strong staining would be assessed a score of 2.3 (0.1×0 + 0.4×2 + 0.5×3 = 2.3). All samples were scored twice independently by two pathologists who were blinded to our study. For PD-L1 and CD47, membranous tumor proportion score (TPS) was applied, during which TPS ≥ 1% and TPS ≥ 5% were set as the positive standard for the two markers, respectively. Co-expression of PD-L1 and CD47 was defined as samples positive in both PD-L1 and CD47. For CD8 and CD68, we calculated the number of CD8-positive TILs and CD68-positive macrophages under six high-power fields and took the average for each specimen.

### Survival Analysis

Overall survival (OS) was used to evaluate the prognosis in both public data and our SqCLC cohort. OS was determined as the time from the diagnosis of SqCLC to the patient’s death, independent from the cause of death. For OS of our cohort, patients who were alive on September 20, 2018, were defined as censored data. The K-M curve was applied to analyze the survival data of patients. We used the R software package “RMS” to portray the Nomogram for patients based on several clinicopathological factors and immune biomarkers, including ERAP2 expression level. In our model, the total point of a patient was generated based on facts from each element and had corresponding 1-year, 2-year, and 3-year survival rates.

### Gene Function Analysis

Based on TCGA (502 samples) and GEO dataset GSE30219 (61 samples) SqCLC transcription data, we used gene enrichment analysis to explore the function of ERAP2-related differentially expressed genes. The enrichment analysis method was described in the previous research (22). The gene enrichment analysis was performed using the following database and gene set: Gene Ontology (GO, c5.all.v7.4.symbols.gmt [Gene ontology]), Kyoto Encyclopedia of Genes and Genomes (KEGG, c2.cp.kegg.v7.4.symbols.gmt [Curated]), Immunologic signatures (c7.all.v7.4.symbols.gmt [Immunologic signatures]). R software was used to visualize the results of gene function enrichment analysis. Based on the above-mentioned TCGA and GEO transcriptome dataset, we classified patients into ERAP2 high and low expression groups using the median ERAP2 expression value and performed differentially expressed genes (DEGs)analysis between them (p<0.05, R package “edgeR” and “limma” were for TCGA data and GEO data, respectively). Then we conducted GO and KEGG enrichment analysis based on the DEGs and drew the bubble charts.

### Immune-Associated Exploration of ERAP2

To identify the potential connection between each two factors of the TMAs, we performed pairwise association analysis. For ERAP2, we classified all SqCLC TMAs patients into high and low expression groups according to the median staining score. The expression of other immune markers was evaluated based on their cut-off values (CD8, CD68: median. PD-L1: 1% and 10%. CD47: 5% and 20%).

We also explored the immune-associated function of ERAP2 in public data using TIMER (http://timer.comp-genomics.org). With this tool, we study the associations between the expression of ERAP2 and several immune markers in SqCLC, including CD68, CD8A, CTLA4, FOXP3, CD274, and CD4. We also explored the relationship between ERAP2 expression and immune cell infiltration levels in SqCLC, including CD8^+^T cell, M0 macrophage, regulatory T cell, M1 macrophage, activated NK cell, and M2 macrophage. CIBERSORT (23) algorithm was applied by TIMER for analysis.

### Statistical Analysis

All data were managed using R software (R x64 4.0.2 Version) and GraphPad Prism 8. An independent-sample was used to make comparisons of continuous and categorical variables between groups. The univariate/multivariate Cox proportional hazard models were used to assess the prognosis. The Kaplan–Meier method was applied to evaluate survival, and the log-rank test was used to determine significance. We used Spearman correlation to explore the associations between variables. We regarded a two-tailed p-value <0.05 as statistically significant.

## Results

### Bioinformatic Mining of ERAP2

We used the GEO dataset GSE30219 (containing SqCLC patients) and classified the patients into better and worse survival groups. DEGs were acquired based on the two groups. According to the p-value and fold-change (FC), we showed 11 DEGs in **Figure 1A**. ERAP2 upregulated in patients with better survival (p-value=0.001, logFC=0.767). According to previous studies, ERAP2 was closely related with CD8 T cell, NK cell, and several immune process (13), indicating it may play important roles in cancer immunology. We then profiled the expression of ERAP2 in multiple cancer types and matched normal tissues by GEPIA2, as shown in **Figure 1B**. We then drew boxplots to show more details on the expression of ERAP2 for NSCLC. We found that ERAP2 expression in SqCLC was statistically lower than that in paired normal tissues (**Figure 1C**), suggesting that the deficiency of ERAP2 might participate in the carcinogenesis of SqCLC. Further, using the GEO dataset GSE30219 (**Figure 1D**) and KM-plotter (http://kmplot.com/analysis/index.php?p=service&cancer=lung) (**Figure 1E**), we drew K-M curves stratified by ERAP2 expression, exhibiting that patients with high ERAP2 expression had longer overall survival in SqCLC (p<0.05).

### Prognostic Roles of ERAP2 in Our Cohort

To validate the prognostic impact of ERAP2 on SqCLC, we established an independent SqCLC cohort, including a total of 190 SqCLC patients, and the tumor samples were retrospectively collected to constitute TMAs. The clinicopathological information of our cohort is shown in **Table 1**. Most patients were males (183, 96.32%) and smokers (176, 92, 63%). According to the 8^th^ AJCC (American Joint Committee on Cancer) Staging Manual (24), there were 34 (17.89%) stage I patients, 70 (36.84%) stage II patients, and 86 (45.26%) stage III patients at diagnosis. At the last follow-up, 112 (58.95%) patients were dead, while the left 78 (41.05%) patients were alive or censored. The median follow-up time was 66.5 months.

**Table 1:** Clinicopathological characteristics of SqCLC patients in tissue microarrays.

Characteristics	Groups	Number of patients (%)
**Gender**	Male	183 (96.32%)
	Female	7 (3.68%)
**Age**	≤60 years old	88 (46.32%)
	>60 years old	102 (53.68%)
**Smoking**	Yes	176 (92.63%)
	No	14 (7.37%)
**T stage**	T1	25 (13.16%)
	T2	99 (52.11%)
	T3	45 (23.68%)
	T4	21 (11.05%)
**N stage**	N0	64 (33.68%)
	N1	67 (35.26%)
	N2	59 (31.05%)
**TNM stage**	I	34 (17.89%)
	II	70 (36.84%)
	III	86 (45.26%)
Tumor diameter*	≤4.5mm	98 (51.58%)
	>4.5mm	92 (48.42%)

*The median of tumor diameter is 4.5mm.

**Figure 2:**
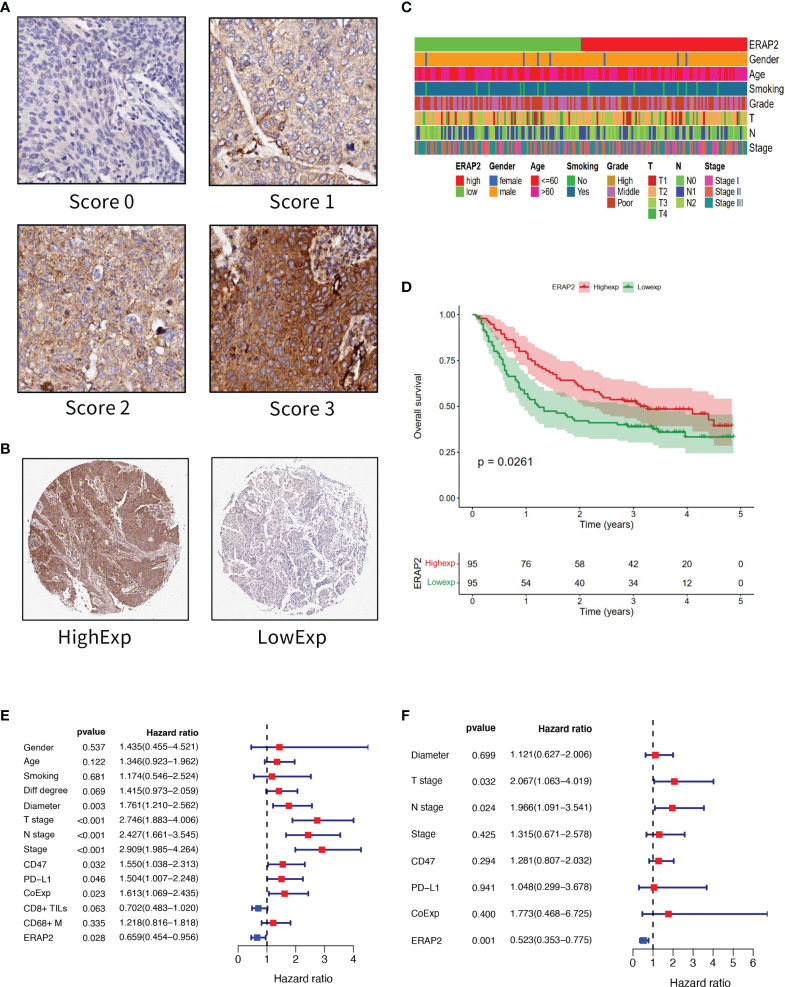
ERAP2 expression in SqCLC tumor microarray (TMA). **(A)** ERAP2 staining intensity scoring standard. ERAP2 expression was graded into 4 categories: 0, 1, 2, and 3 according to the staining intensity. The larger number represented higher ERAP2 staining intensity (100×). The final score of samples is evaluated by multiplying the grade and corresponding area percentages. **(B)** Example of high and low ERAP2 expression in TMA. Left, high expression sample. Right, low expression sample (20×). **(C)** Clinicopathological information related heatmap of SqCLC TMA. **(D)** K-M curve showing the overall survival in SqCLC TMA stratified by ERAP2 expression. Red, ERAP2 high expression group. Green, low expression group. **(E)** Univariate analysis forest plot of clinicopathological factors, ERAP2 expression, and other immune markers’ expression in SqCLC TMA. CoExp, PD-L1, and CD47 co-expression. CD8+ TILs, CD8+ tumor infiltration lymphocytes. CD68+ M, CD68+ infiltration macrophages infiltration level. The red square represents risk factors for prognosis, while the blue square represents protective factors for patients. **(F)** Multivariate analysis forest plot of factors, which could predict the overall survival in the univariate analysis. CoExp, PD-L1, and CD47 co-expression. CD8+ TILs, CD8+ tumor infiltration lymphocytes. The red square represents risk factors for prognosis, while the blue square represents protective factors for patients.

IHC of ERAP2 on our TMAs was conducted, and we calculated an IHC score for each specimen as the sum of the percentage of stained cells multiplied by the staining intensity. The standard of staining intensity was displayed in **Figure 2A**. The median IHC score of ERAP2 is 1.375, and we classified the samples into high and low ERAP2 expression subgroups using the median score as the cut-off value. Representative images of high expression and low expression of ERAP2 were shown in **Figure 2B**. We profiled the demographic information for each patient annotated for the expression level of ERAP2, and the distributions of characteristic features did not show a noticeable difference between ERAP2 high and low expression subgroups (**Figure 2C**). K-M curve exhibited that in our cohort, patients with ERAP2 high expression had significantly longer overall survival than the ERAP2 low expression subgroup (**Figure 2D**).

To further explore the prognostic marker of SqCLC, firstly we performed a univariate analysis in our cohort (**Figure 2E** and **Table 2**). Then we conducted the multivariate analysis using the variables that were proved to be statistically significant in univariate analysis (**Figure 2F** and **Table 3**). ERAP2 was identified as an independent protective factor for SqCLC (Univariate, HR =0.659, 95% CI, 0.454-0.956, p-value=0.028; Multivariate, HR =0.523, 95% CI, 0.353-0.775, p value=0.001). The ERAP2 score and clinical information of each sample in our cohort was recorded in **Supplementary Data 1**.

**Table 2:** Univariate Cox Regression of Prognostic Factors in SqCLC.

	OS	
	HR (95% CI)	p
Age		0.122
≤60y	1.000	
>60y	1.346 (0.923-1.962)	
Gender		0.537
Female	1.000	
Male	1.435 (0.455-4.521)	
Smoking		0.681
No	1.000	
Yes	1.174 (0.546-2.524)	
Tumor Diameter		0.003
≤median	1.000	
>median	1.761 (1.210-2.562)	
T stage		1.56E-07
T1+T2	1.000	
T3+T4	2.746 (1.883-4.006)	
N stage		0.000
N0+N1	1.000	
N2	2.427 (1.661-3.545)	
TNM Stage		0.000
I+II	1.000	
III	2.090 (1.985-4.264)	
CD47 expression		0.032
Low	1.000	
High	1.55 (1.038-2.313)	
PD-L1 expression		0.046
Negative	1.000	
Positive	1.504 (1.007-2.248)	
PD-L1 and CD47 co-expression		0.023
No	1.000	
Yes	1.163 (1.069-2.435)	
CD8+ TILs level		0.063
Low	1.000	
High	0.702 (0.483-1.02)	
CD68+Macrophage infiltration level		0.335
Low	1.000	
High	1.218 (0.816-1.818)	
ERAP2 expression		0.028
Low	1.000	
High	0.659 (0.454-0.956)	

All p values were two sides and less than 0.05 were considered significant.

SqCLC, Squamous cell lung cancer; OS, overall survival; HR, hazard ratio; CI, confidence interval.

Low and high expression was classified as the median except for CD47 (1% as cut-off value).

**Table 3:** Multivariate Cox Regression of Prognostic Factors in SqCLC.

	OS	
	HR (95% CI)	p
Diameter		0.699
≤60y	1.000	
>60y	1.121 (0.627-2.006)	
T stage		0.032
T1+T2	1.000	
T3+T4	2.067 (1.063-4.019)	
N stage		0.024
N0+N1	1.000	
N2	1.966 (1.091-3.541)	
TNM stage		0.425
I+II	1.000	
III	1.315 (0.671-2.578)	
CD47 expression		0.294
Negative	1.000	
Positive	1.281 (0.807-2.032)	
PD-L1 expression		0.941
Negative	1.000	
Positive	1.048 (0.299-3.678)	
PD-L1 and CD47 co-expression		0.400
No	1.000	
Yes	1.773 (0.468-6.725)	
ERAP2 expression		0.001
Low	1.000	
High	0.523 (0.353-0.775)	

All p values were two sides and less than 0.05 were considered significant.

SqCLC, Squamous cell lung cancer; OS, overall survival; HR, hazard ratio; CI, confidence interval.

Low and high expression was classified as the median except for CD47 (1% as cut-off value).

### Construction of Nomogram

**Figure 3:**
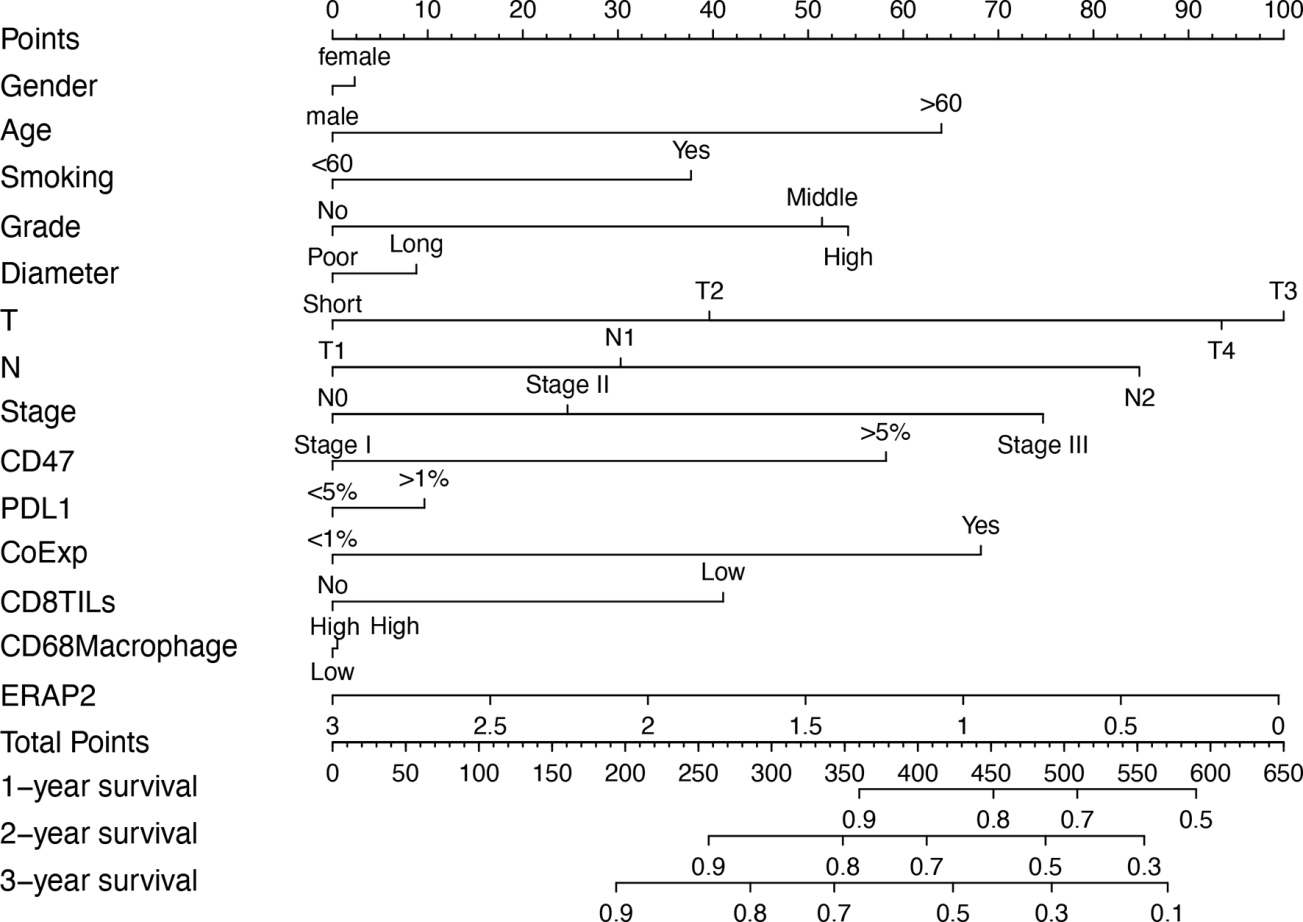
Prognostic nomogram based on the clinicopathological factors, ERAP2 expression, and other immune markers predicting the 1-year, 2-year, and 3-year overall survival rate in our SqCLC cohort. T, T stage. N, N stage. CoExp, PD-L1and CD47 co-expression. CD8TILs, CD8+ tumor infiltration lymphocytes. CD68 Macrophage, CD68+ infiltration macrophages.

The 190 SqCLC patients with complete clinical information and follow-up survival time from our cohort were used to establish a prognostic nomograph using R software with the RMS package (**Figure 3**). All clinicopathological factors and immune markers expression status were included in the nomogram model. In the nomogram, the ERAP2 expression was a decisive parameter among all immune markers, for which score 0 represented 100 points and scored three indicated 0 points. The T stage behaved like a very effective indicator among clinicopathological factors, and T3 represented 100 points. The weight of gender, tumor diameter, PD-L1, and CD68+ macrophages were relatively low compared to other indicators. Total points over 700 could predict a survival rate <50% in 1-year survival, a survival rate <20% in 2-year survival, and a survival rate <10% in 3-year survival.

### Exploration of ERAP2-Related Function by Pathway Enrichment

**Figure 4:**
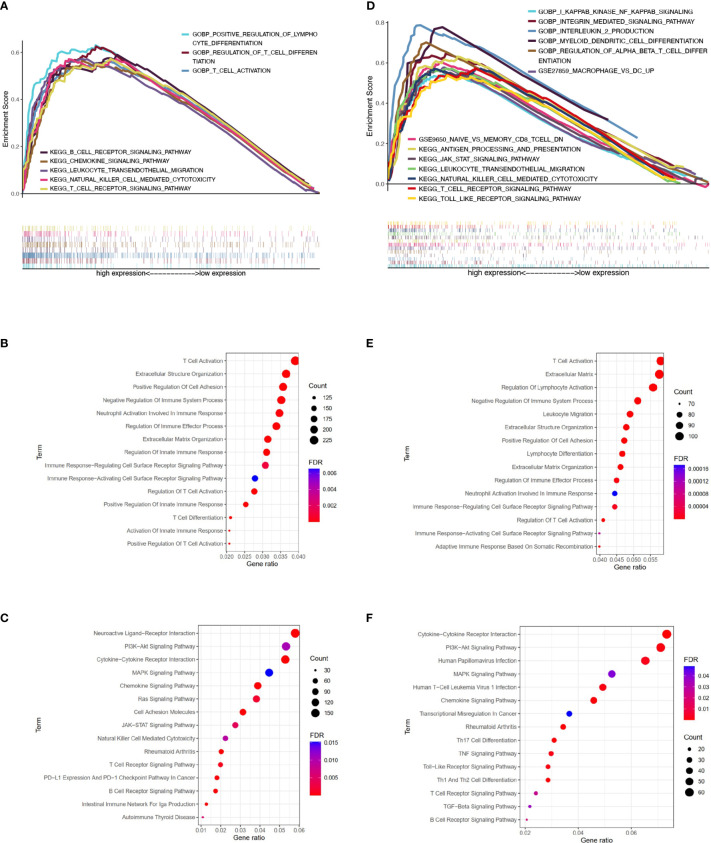
Pathway enrichment analysis of patients with different ERAP2 expression level in SqCLC public data. **(A)** Enrichment analysis based on TCGA SqCLC data. Upper: pathways, terms, or gene sets enriched in ERAP2 high expression group. **(B)** Representative GO terms and pathways enriched in DEGs between high and low ERAP2 expression groups based on TCGA SqCLC data. **(C)** Representative KEGG terms and pathways enriched in DEGs between high and low ERAP2 expression groups based on TCGA SqCLC data. **(D)** Enrichment analysis based on GEO SqCLC data. Upper: pathways, terms, or gene sets enriched in ERAP2 high expression group. **(E)** Representative GO terms and pathways enriched in DEGs between high and low ERAP2 expression groups based on GEO SqCLC data. **(F)** Representative KEGG terms and pathways enriched in DEGs between high and low ERAP2 expression groups based on GEO SqCLC data.

Based on SqCLC data from TCGA (**Figures 4A**–**C**) and GEO (**Figures 4D**–**F**), we analyzed signaling pathway enrichment for patients with ERAP2 expression levels. We revealed that high ERAP2 expression was synergistic with several immune-promoting biological processes. In both TCGA and GEO databases, high ERAP2 expression was enriched in the natural killer cell-mediated cytotoxicity pathway and T cell receptor signaling pathway, consistent with previous studies. In the TCGA database, high ERAP2 expression was also enriched in the positive regulation of lymphocyte differentiation, T cell activation terms, and positive regulation of innate immune response, suggesting that high ERAP2 expression was correlated with the immune active environment. In the GEO database, high ERAP2 expression was enriched in the PI3K-Akt signaling pathway, JAK-STAT pathway, and the Toll-like receptor pathway, arousing our interest in the molecular network about ERAP2.

### Immune-Related Exploration of ERAP2 in Public Data

**Figure 5:**
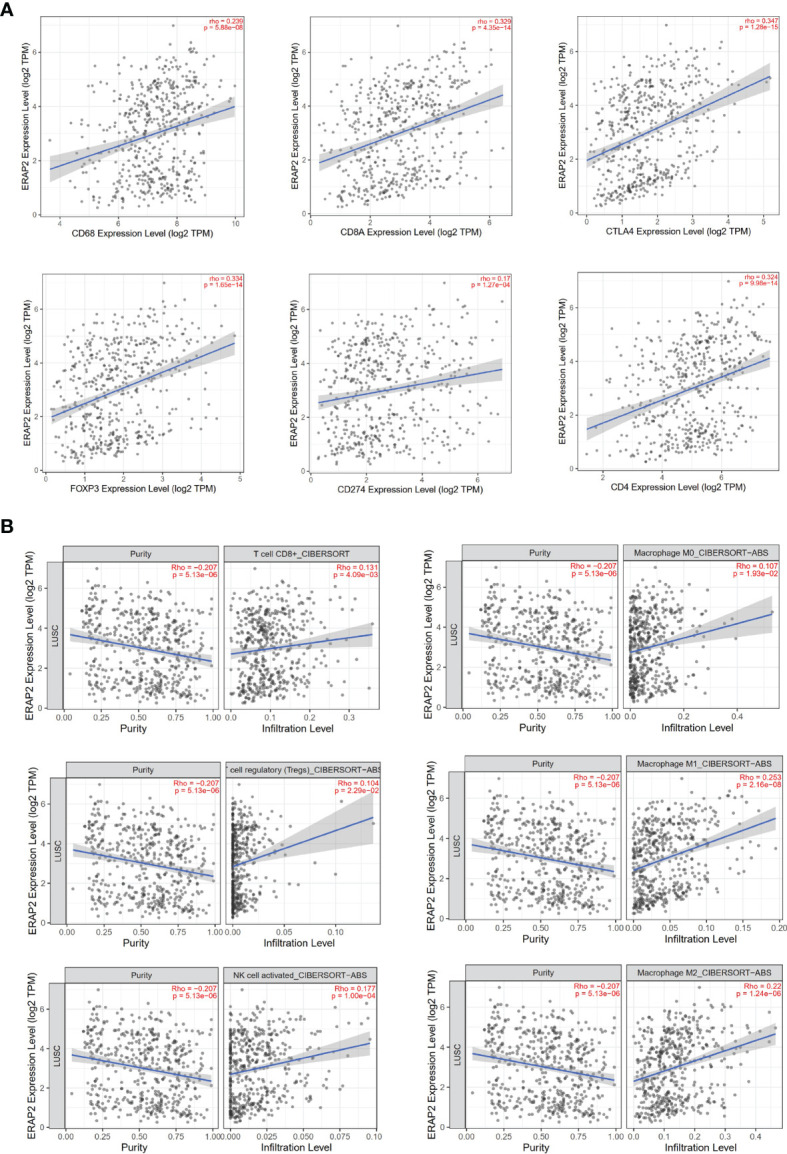
Immune associations about ERAP2 expression using public data (TIMER). **(A)** Correlations between the expression of ERAP2 and immune markers in SqCLC. Top: CD68, CD8A, CTLA4. Bottom: FOXP3, CD274, CD4. The correlation coefficient and p-value are in the top right corner of each picture. **(B)** Correlations between ERAP2 expression and immune cell infiltration level in SqCLC. Top CD8+ T cell, M0 macrophage. Middle: regulatory T cell regulatory, M1 macrophage. Bottom: Activated NK cell, M2 macrophage.

Inspired by the pathway enrichment analysis results, we further explored the correlations between the expression of ERAP2 and several immune markers in public data using TIMER (an online tool). In **Figure 5A**, we showed the association between the expression level of ERAP2 and CD68, CD8A, CTLA4, FOXP3, CD274 (PD-L1), and CD4, in which ERAP2 was positively correlated with these markers and all the correlations were statistically significant (p=5.88e-08 for CD68, p=4.35e-14 for CD8A, p=1.28e-15 for CTLA4, p=1.65e-14 for FOXP3, p=1.27e-04 for CD274, p=9.98e-14 for CD4), indicating that ERAP2 may enhance the antitumor immune response.

We then characterized the interactions between ERAP2 expression and immune cell infiltration levels in SqCLC samples (**Figure 5B**). Several immune cells were selected, including CD8^+^ T cells, M0 macrophages, regulatory T cells, M1 macrophages, activated NK cells, and M2 macrophages. ERAP2 expression was significantly positively correlated to the infiltration levels (CD8^+^ T cells, p=4.09e-03; M0 macrophages, p=1.93e-02; regulatory T cells, p=2.29e-02; M1 macrophages, p=2.16e-08; activated NK cells, p=1.00e-04; M2 macrophages, p=1.24e-06), consistent with previous findings in our study, further suggesting that ERAP2 may act as a tumor suppressor in SqCLC in an immune-promoting manner.

### Immune-Related Exploration of ERAP2 in Our Cohort

**Figure 6:**
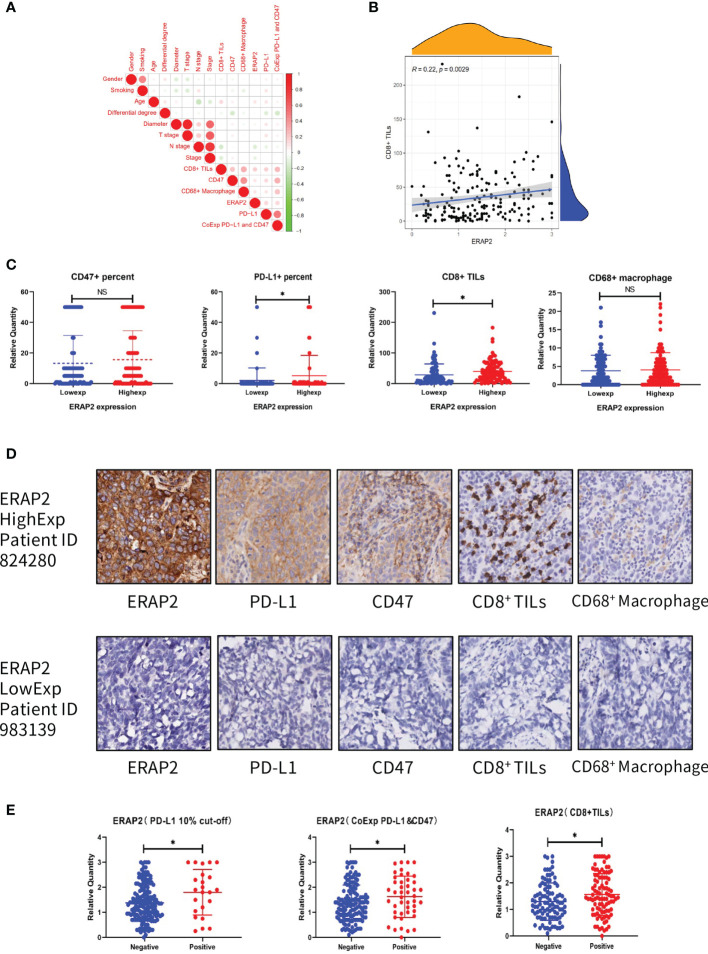
Association between ERAP2 expression and multiple immune markers in our SqCLC TMA. **(A)** Associations among all clinicopathological and immune markers in SqCLC TMAs. The circle color represents the correlational tendency. The circle size represents the statistical significance. Red, positive correlation. Green, negative correlation. Larger circle represents the lower p-value. **(B)** Correlation between ERAP2 expression and CD8+ TILs. Spearman relevant coefficient = 0.22, p-value=0.0029. **(C)** The relative quantity of immune markers in ERAP2 high and low expression patients of SqCLC TMAs. From left to right: CD47, PD-L1, CD8, and CD68. Blue, ERAP2 low expression group. Red, ERAP2 high expression group. Asterisk, p-value<0.05. **(D)** IHC images of representative patients in the ERAP2 high and low expression group (100×). Upper: representative patient with high ERAP2 expression. Lower: representative patient with low ERAP2 expression. From left to right: ERAP2, PD-L1, CD47, CD8^+^ TILs and CD68^+^ Macrophages. **(E)** The relative quantity of ERAP2 among patients with different immune marker statuses. From left to right: PD-L1, PD-L1 and CD47 co-expression, CD8+ TILs. Asterisk, p-value < 0.05.

We evaluated the IHC expression level of PD-L1, CD47, CD8, and CD68 in our cohort, and as shown in **Figure 6A**, pairwise correlation analysis was performed for all clinicopathological factors and molecular markers. We found that ERAP2 expression was significantly correlated with CD8^+^ TILs (Spearman r=0.22, p=0.0029, **Figure 6B**). Applying the median staining score of ERAP2 expression as the cut-off value (median=1.375), we classified all 190 SqCLC patients of our cohort into two groups and compared the expression levels CD47 and PD-L1 and the infiltrating density of CD8^+^ TILs and CD68^+^ Macrophages of them (**Figure 6C**). In the ERAP2 high expression group, the expression level of PD-L1 was significantly higher (p<0.05), meanwhile, the infiltrating density of CD8^+^ TILs were also considerably higher in the ERAP2 high expression group (p<0.05). ERAP2 expression in positive groups was significantly higher than in negative groups for all three immune markers, implying ERAP2 may exert significant functions in anti-tumor immunity. The representative images of each one patient from the ERAP2 high and low expression group (**Figure 6D**). In addition, we distributed all the patients of our cohort into positive and negative groups according to the status of PD-L1 (10% TPS as cutoff value), the status of PD-L1/CD47 co-expression (co-expression criteria: 1% for PD-L1 and 5% for CD47) and infiltrating density of CD8^+^ TILs (median as cutoff value). We compared the ERAP2 expression levels between the groups (**Figure 6E**).

## Discussion

SqCLC has been a refractory disease for decades, with few inroads in targeted therapy, and only a minority of SqCLC patients have achieved sustained benefits from immunotherapy (5, 25). Based on public databases and our SqCLC cohort (190 patients), our study identified ERAP2 as a favorable prognostic indicator and potential enhancer for ICIs in SqCLC.

It is reasonable for us to believe that ERAP2 may exert a significant role in SqCLC. Using GEO and TCGA data, we found ERAP2 was lowly expressed in SqCLC and was significantly associated with longer survival. As an endoplasmic reticulum-settled enzyme, ERAP2 is responsible for the final trimming of peptides represented by the major histocompatibility complex (MHC) class I molecules (13). Previous researcher has demonstrated that ERAP2 enzyme function could affect T cell and NK cell responses towards normal and cancer cells as well as the synthesis of inflammatory cytokines (26). Since ERAP2 is deeply involved in the generation and destruction of immunopeptidome, its deficiency can lead to disorders in anti-tumor immunity activation. Considering our findings in the public database, we speculated that ERAP2 is of great worth in SqCLC.

We are the first to illustrate the predictive value of ERAP2 in SqCLC. The predictive value of ERAP2 was validated in SqCLC TMAs with high quality. All patients in our cohort accepted no pre-surgery treatments, and the most recent patient was diagnosed in 2011, offering a long enough follow-up period. In our cohort, ERAP2 expression was positively related to overall survival, consistent with what we found in the public database, providing further evidence for the definition of ERAP2 as a prognostic indicator in SqCLC. Moreover, using multivariate analysis, we confirmed that ERAP2 expression was an independent prognostic factor for SqCLC individuals based on our cohort.

Based on previous research, we hypothesized that the positive predictive function of ERAP2 in SqCLC derived from its ability to prevent immune evasion by modulating immune recognition (27) because improper trimming of peptides in ER is one of the strategies that cancer cells avoid the attack from the immune system (28). In 2021, Mpakali et al. (14) reported that ERAP2 can trim the peptides that enter the ER and are too long to fit into MHC I molecule. However, while trimming peptides, ERAP2 can also destroy tumor-associated antigenic peptides destined for loading on MHC I, thus affecting avoiding T cell response.

Little was known about how ERAP2 behaves in lung cancer. In 2014, Zhou et al. (29) reported that ERAP2 rs2248374/rs2549782-AG haplotype was significantly associated with increased NSCLC risk, while ERAP2 rs2248374/rs2549782-GT haplotype individuals tended to indicate a reduced risk. In 2021, Wis ´niewski et al. (30) asserted that the extent of ERAP2 presence could affect the anti-cancer response of ERAP1 in NSCLC. To our knowledge, there has been no study focusing on the ERAP2 expression in lung cancer so far, and the area of ERAP2 in SqCLC has never been set foot in.

One of the highlight conclusions of our study is that in both public data and our cohort, high expression of ERAP2 is correlated with the immunoreactive tumor microenvironment, which is favorable for administering immunotherapy in SqCLC. Due to its malignant nature and intricate genomic architecture, many SqCLC patients are marginalized in the prosperity of immunotherapy, failing to obtain sustained benefits from ICIs (31). In the realm of SqCLC, methods that can boost immunotherapy efficacy are demanding. The tumor microenvironment (TME) is an integral component of cancer, composed of various cell types crucial to tumor immunology (32). The TME infrastructure and the interactions between cancer cells and TME during cancer initiation and progression could dictate the response to immunotherapy (33). In our study, high ERAP2 expression was associated with high levels of multiple immune markers and cells, including PD-L1, CD47, CD8^+^ TILs, CD68^+^ Macrophages, and NK cells which were positive indicators for immunotherapy. Our findings of ERAP2 and NK cells were consistent with previous studies (13, 15), while we are the first to illustrate the associations between ERAP2 and other immune cells and markers. The positive association between ERAP2 and FOXP3 aroused our interest. Although FOXP3 was previously reported to promote tumor growth and metastasis in NSCLC (34), there were also researches asserting that tumoral FOXP3 had the potential to suppress tumor function in SqCLC (35). The involvements between FOXP3 and tumor immunity have not been clearly illustrated, and current results of tumor FOXP3 are inconsistent and inconclusive (36). The ERAP2 high expression group harbored an immune-active TME, suggesting the feasibility of immune-related treatments among SqCLC with ERAP2 high expression and the potentiality of ERAP2 as a biomarker for SqCLC immunotherapy. ERAP2 was also involved in autoimmune diseases, such as ankylosing spondylitis (34), whose etiology is unclear. The immune activation process during pre-eclampsia was also reported to be engaged with the differential expression of ERAP1/2 (37), suggesting the importance of ERAP2 in the immune system (38).

Under the pandemic of SARS-CoV-2, ERAP2 abnormity is related to the unfavorable clinical outcomes of COVID-19 infected patients, while ERAP2 may also inspire the development of the vaccine. Recently, ERAP2 was reported to participate in the virus antigen presentation process of COVID-19 (39, 40). ERAP2 and its homolog ERAP1 constituted an efficient filter to epitope presentation by greatly limiting the diversity of virus antigenic peptides sequences produced, suggesting the promising value of ERAP2 in SARS-CoV-2 immunogenicity studies and vaccine design (41). The rs150892504 mutations in the ERAP2 gene were believed to be a genetic factor related to severe life-threatening complications in individuals infected with coronavirus (42). Since ERAP2 was involved in the renin-angiotensin system (RAS), ERAP2 dysfunction was hypothesized to exacerbate the symptomatology and prognosis of the SARS-CoV-2.

Several limitations existed in our study. First, our TMAs only contained tumor tissue of each patient, lacking the data of matched normal samples. Secondly, we only performed IHC to a limited number of immune markers, which were far from enough to represent the whole ecosystem of TME. Third, we did not validate our findings at the transcriptional level because there were no fresh or fresh frozen samples.

In conclusion, our study is the first to intensively illustrate the role of ERAP2 in lung cancer. We identified ERAP2 as a positive prognostic biomarker for SqCLC and revealed its potentiality in predicting immunotherapy response, offering novel ideas for the administration of ICIs in SqCLC.

## Footnote

The authors are accountable for all aspects of the work in ensuring that questions related to the accuracy or integrity of any part of the word are approximately investigated and resolved.

Written informed consents for the publication of details relating to any participant were obtained from that person.

## Data Availability Statement

The raw data supporting the conclusions of this article will be made available by the authors, without undue reservation.

## Ethics Statement

The studies involving human participants were reviewed and approved by the Ethics Committee of National Cancer Center/Cancer Hospital, Chinese Academy of Medical Sciences, and Peking Union Medical College. The patients/participants provided their written informed consent to participate in this study.

## Author Contributions

JH, SG, and CL were responsible for conception and design. JH, SG, JW, CL, and BZ offered administrative support. HT and ZY provided study materials and clinical resources. HT, FB, and ZZ were responsible for the collection and assembly of data. HT, ZY, FB, and JY were responsible for data analysis. JX, RL, YP, GB, YT, YC, LL, TF, CX, and YZ were responsible for data interpretation. All authors were contributed to the writing and final approval of the manuscript.

## Funding

This work was supported by the National Natural Science Foundation of China (No: 81972196, 8210103136, 82060426, 81702274); the National Key R&D Program of China (2020AAA0109505); the Beijing Municipal Science & Technology Commission (Z191100006619119); the Special Research Fund for Central Universities, Peking Union Medical College (3332021028, 3332021029); Beijing Hope Run Special Fund of Cancer Foundation of China (LC2018B14, LC2020B09).

## Conflict of Interest

Author JY was employed by company Genetron Health (Beijing) Co. Ltd. 

The remaining authors declare that the research was conducted in the absence of any commercial or financial relationships that could be construed as a potential conflict of interest.

## Publisher’s Note

All claims expressed in this article are solely those of the authors and do not necessarily represent those of their affiliated organizations, or those of the publisher, the editors and the reviewers. Any product that may be evaluated in this article, or claim that may be made by its manufacturer, is not guaranteed or endorsed by the publisher.

## Supplementary Material

Supplementary Data Sheet 1 The clinicopathological data of Tissue Microarrays.
